# Ocular Hypertension after Pediatric Cataract Surgery: Baseline Characteristics and First-Year Report

**DOI:** 10.1371/journal.pone.0069867

**Published:** 2013-07-29

**Authors:** Haotian Lin, Weirong Chen, Lixia Luo, Xinyu Zhang, Jingjing Chen, Zhuoling Lin, Bo Qu, Jiao Zhan, Danying Zheng, Xiaojian Zhong, Zhen Tian, Yizhi Liu, for the Study Group of CCPMOH

**Affiliations:** State Key Laboratory of Ophthalmology, Zhongshan Ophthalmic Center, Sun Yat-sen University, Guangzhou, China; Bascom Palmer Eye Institute, University of Miami School of Medicine, United States of America

## Abstract

Monitoring intraocular pressure (IOP) is essential for pediatric cataract treatment but always difficult due to lack of cooperation in young children. We present the baseline characteristics and the first-year results of a long-term prospective cohort study, which are aimed to determine the relationship of the incidence of ocular hypertension (OH) in children after cataract surgery during the first-year period and the risk of developing late-onset glaucoma. Children were included with the following criteria: they were≤10 years old and scheduled to undergo cataract surgery with/without intraocular lens implantation; they were compliant with our follow-up protocol, which included monitoring IOP using a Tono-Pen under sedation or anesthesia. Incidence of OH, peak OH value, OH onset time and OH duration within a 12-month period following surgery were measured. In brief, 206 patients (379 eyes) were included and OH developed in 66 of 379 (17.4%) eyes. The mean follow-up period was 14.0±3.2 months (median, 12 months; range, 10–16 months). Moreover, 33 of 196 (16.8%) aphakic eyes and 33 of 183 (18.0%) IOL eyes were diagnosed with OH. The peak OH onset times were at 1-week (34/66, 51.5%) and 1-month (14/66, 21.2%) appointments postsurgery. The peak IOP value in the OH eyes was 29.9±7.5 mmHg (median, 29 mmHg; range, 21–48 mmHg). The duration of OH was 30.9±31.2 days (median, 30 days; range, 3–150 days). OH recurred in 13 eyes with a history of OH diagnosed within 1 month postsurgery (13/54, 24.1%), which needed temporary or long term use of antiglaucoma medications. In conclusion, the incidence of OH in children after cataract surgery was 17.4% during the first-year period. Children who have suffered elevated IOP in the first year after cataract surgery should be followed closely to determine if there is an increased risk of developing late-onset glaucoma.

## Introduction

Ocular hypertension (OH) and glaucoma are significant postoperative complications of pediatric cataract surgery, but their causes are complex and not fully understood. [1] OH, which is defined as intraocular pressure (IOP) greater than 21 mm Hg, and secondary glaucoma, which is defined as a particular pattern of optic nerve and visual field damage, both have been reported with a wide frequency during long-term follow-up of pediatric cataract surgery patients. [2]–[4] It has been hypothesized that OH is a risk factor for glaucoma and years of OH might precede a late-onset glaucoma diagnosis [1], [5].

Most previously reported studies have focused on late-onset OH and glaucoma, and it has been reported that the rate of progression from late-onset OH to glaucoma is 23% based on 5- and 10-year examinations. [1] However, few studies have addressed the elevation in IOP in the first year following pediatric cataract surgery and the significance of the first-year elevation in IOP to late-onset glaucoma. Moreover, elevated IOP and increased fluctuation of IOP have been determined to be factors for glaucoma progression in an early-stage glaucoma trial in adults. [4] It has been hypothesized that many factors, such as surgical inflammation and/or steroid response, [5]–[12] would elevate IOP and cause OH immediately after pediatric cataract surgery, which may differ from the time frame of late-onset OH that has been previously reported [1]–[3].

Until now, there are few clinical studies regarding early-onset IOP elevation in a cohort of children following cataract surgery who were routinely treated with topical corticosteroids [4]–[6]. Furthermore, the risk of developing late-onset glaucoma for those children who have suffered elevated IOP in the first year postsurgery is unknown and there is still lack of study regarding the early signs and early intervention of these possible-high-risk patients. In this study, we report the first-year result of monitoring IOP in children after cataract surgery, in which we aimed to determine the incidence of OH in a cohort of the included children, and to present the baseline characteristics for an ongoing prospective cohort study to ultimately address the significance of the first-year elevation in IOP to late-onset glaucoma.

## Subjects and Methods

### Patients

Pediatric patients were selected from the Childhood Cataract Program of the Chinese Ministry of Health (CCPMOH), which includes a series of ongoing studies on the influence of early interventions on long-term outcomes of pediatric cataract treatment. [13] The study was approved by the institutional review board of Zhongshan Ophthalmic Center (ZOC), Guangzhou, China. Informed written consent was obtained from at least one parent of each participating child, and the tenets of the Declaration of Helsinki were followed throughout this study.

**Figure 1:**
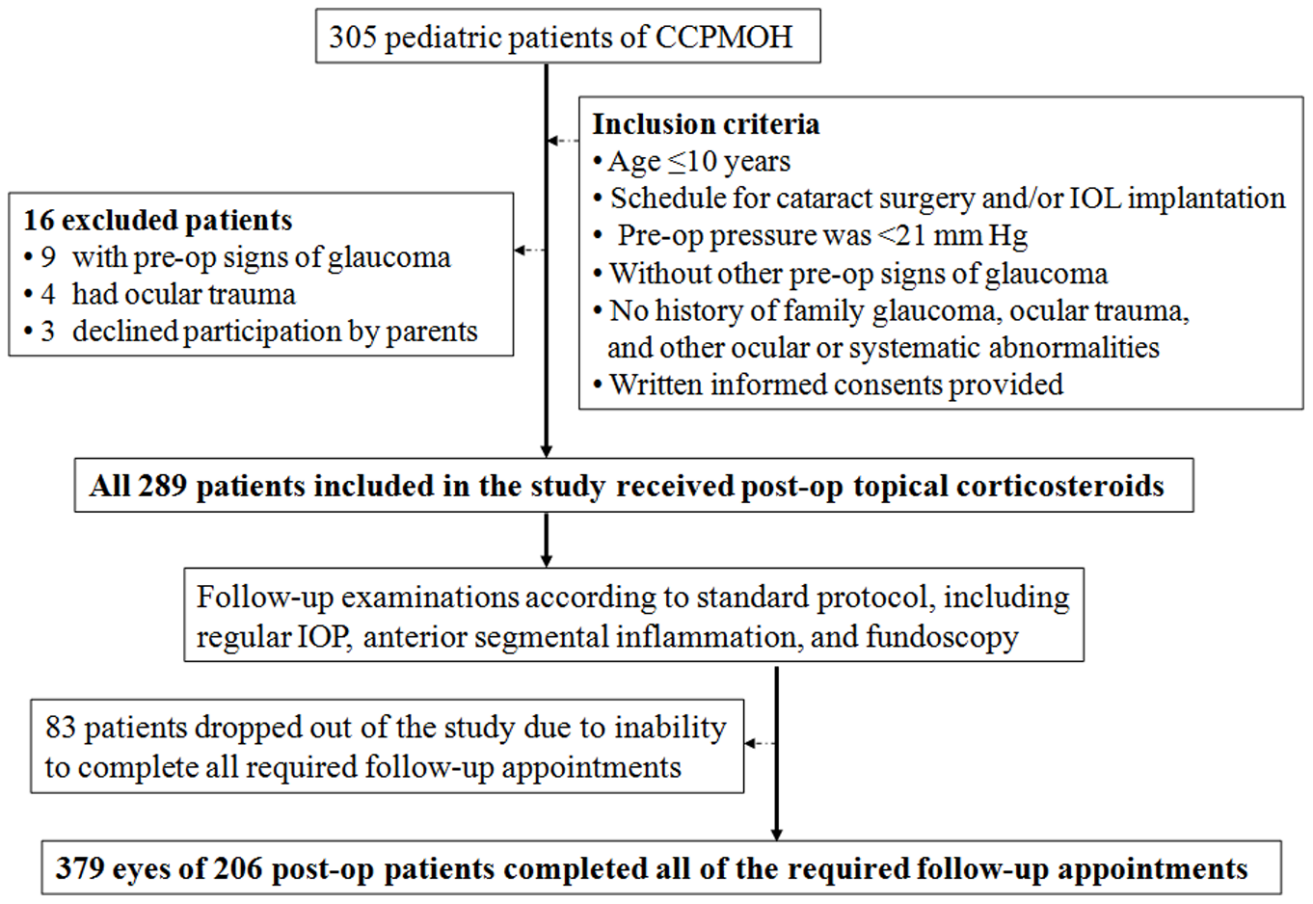
Flow chart for patient selection and follow-up protocol. (Notes: CCPMOH = Childhood Cataract Program of the Chinese Ministry of Health; IOL = intraocular lens; OH = ocular hypertension).

All pediatric cataract patients who were younger than 10 years old, registered in the CCPMOH and had undergone cataract surgery and/or intraocular lens (IOL) implantation at ZOC between December 2010 and April 2012 were included. Eyes were excluded if their preoperative pressure were >21 mm Hg or if there were other preoperative signs of glaucoma, such as corneal enlargement, corneal clouding, or excessive optic nerve cupping (as judged by the examining physician or as documented by an optic nerve cup-to-disk ratio >0.4 or by an asymmetry between eyes >0.2), family glaucoma history, ocular trauma, or other abnormalities, such as microcornea, persistent hyperplastic primary vitreous, rubella or Lowe syndrome. This relatively low intraocular pressure exclusion criterion was chosen to limit the possibility of including patients with presurgery glaucoma. All included patients were required to complete all of the follow-up appointments that our protocol specified. Those patients who were noncompliant with our follow-up protocol for assessment of the incidence of OH (defined in the following) were excluded. The flow chart for patient selection and follow-up during the first-year period was shown in Figure 1.

### Surgical Technique

Cataract surgery with/without IOL implantation was performed by two equivalently experienced pediatric ophthalmologists (YZL and WRC). Variation in the surgical technique was a function of the patient’s age, the year of surgery and the degree of posterior capsule opacity. A limbal or scleral tunnel incision was employed. An anterior capsulotomy was made in a continuous curvilinear fashion. The nucleus and cortex were removed using a manual irrigation/aspiration device or an automated vitrectomy instrument. The vitrectomy instrument was used to create a central posterior capsulotomy and perform a limited anterior vitrectomy in select cases. Peripheral iridectomy was rarely performed. Those patients undergoing primary implantation of an intraocular lens or kept as aphakic eyes were included in the study. All of the surgeries were performed under general anesthesia.

### Postoperative Medicine Regimen

The standard postoperative regimen consisted of 2 mg of subconjunctival dexamethasone and tobradex eye drops (tobramycin 0.3%, dexamethasone 0.1%, Alcon) 4 times per day and tobradex eye ointment (tobramycin 0.3%, dexamethasone 0.1%, Alcon) once per night for 1 month. Subsequently, anti-inflammatory steroid drugs were replaced with a steroid-free drug administration for another month (pranoprofen eye drops, 4 times per day, Senju Pharmaceutical Co., Ltd. JP). In all OH cases, once an OH eye was diagnosed, topical steroid administration was discontinued and replaced with pranoprofen eye drops. However, additional antiglaucoma drugs were administered according to the following regimen: if the IOP in the OH eye was <25 mmHg, steroid use only would be discontinued. If the IOP was between 25 and 30, one antiglaucoma drug (2% carteolol eye drops, 2 times per day) would be administered. If the IOP was between 30 and 40, two types of antiglaucoma drugs (2% carteolol eye drops, 2 times per day and 1% brinzolamide eye drops, 2 times per day) would be administered. If the IOP was ≥40, three types of antiglaucoma drugs (2% carteolol eye drops, 2 times per day; 1% brinzolamide eye drops, 2 times per day and 0.2% brimonidine eye drops 2 times per day) would be administered. Antiglaucoma drugs regimen were administered two more weeks after the IOP was controlled in the normal range, then the drugs were gradually decreased and discontinued in another two weeks.

### Follow-up Protocol

The follow-up protocol was consistent with our previous study [13]. However, additional follow-up appointments were made for the following reasons: OH was diagnosed, the standard postoperative regimen of steroid administration was discontinued, and/or antiglaucoma medications were added. In those cases, the rescheduled follow-up protocol specified visits at 3 days, 1 week, 1 month, 2 months and 3 months and subsequently, every 3 months after an OH diagnosis within a 12-month period postsurgery. Children with IOP that could not be controlled within 1 week were monitored with consultation and assistance from the ZOC glaucoma department.

### Definition of OH and IOP Assessment

OH was defined as IOP>21 mmHg that was first diagnosed in the follow-up appointments within a 12-month period postsurgery. We defined OH as IOP>21 mmHg to allow comparisons with previous studies that used the same definition [9]–[11], [14].

Because infants and young children have poor compliance with examination, we developed techniques and equipment specific for pediatric ophthalmic examination and have obtained an international patent (Patent No. US-2013-0074265-A1, the specific techniques not shown, for query please input 20130074265 to http://appft.uspto.gov/netahtml/PTO/srchnum.html ). To assess IOP, we performed examinations immediately after administering a sedative drug (i.e., chloral hydrate as a sleep aid) or under general anesthesia (if chloral hydrate was contraindicated or unacceptable) for children who were uncooperative to allow for regular examinations in the clinic.

IOP was regularly measured on every follow-up appointment and were confirmed at least twice using a Tono-Pen (Reichert Inc., Seefeld, Germany) by two different pediatric ophthalmologists to rule out operational error. It was usually possible to measure IOP in infants using a Tono-Pen tonometer under sedation or anesthesia, whereas older and cooperative children tolerated a Goldman tonometry or pneumotonometry better, which were simultaneously used to confirm Tono-Pen results.

### Outcome Measure during the First-year Period

The primary outcome measure was the incidence of OH, which was based on the IOP examination and definition of OH. The secondary outcome measures were OH onset (diagnosis) time after surgery, peak OH value, OH duration (the time between OH appearance and disappearance without considering whether medications were used), and the number of OH children with symptoms.

### Statistical Analysis

Demographic and clinical information was recorded at baseline. Statistical analyses were performed for each patient eye using a chi-squared test for categorical variables and Student *t*-test for continuous variables with SPSS (Version 17.0; SPSS, Inc., Chicago, IL). The criterion for significance was P<0.05. To adjust for potential intrasubject correlation in the Cox models, the robust sandwich variance estimate of Lin and Wei was used. [15] Associations of incidence rates across sex, cataract laterality, status after treatment, and age groups were tested by the Mantel-Haenszel trend test.

## Results

In all, 379 eyes of 206 (206/305, 67.5%) children (143 male and 63 female) were included in this study and completed all of the required follow-up appointments from the CCPMOH. The primary reasons for exclusion were pre-op signs of glaucoma (9/305, 3.0%), ocular trauma (4/305, 1.3%), refusal to participation by parents (3/305, 1.0%) and 83 patients dropped out of the study due to inability to complete all required follow-up appointments, which were depicted in Figure 1. Descriptive data for the study numbers of patients and eyes as related to the incidence of post-operative OH are presented in Table 1. We found an apparent cut-off age at surgery (median, 36 months; range, 4–120 months) for the development of OH at 12 months (overall children) by the Mantel-Haenszel trend test (P<0.05). 23.6% (30/127) of patients who underwent surgery and were 12 months old or younger developed OH, whereas 14.3% (36/252) of patients older than 12 months developed OH. Of 173 patients with bilateral cataracts who fulfilled the study eligibility criteria for both eyes, OH developed in at least one eye in 41 (23.7%) patients; in 20 (48.8%) of these patients, OH occurred bilaterally. Of 33 patients with unilateral cataracts, OH developed in 5 (15.2%) patients. In patients with a unilateral cataract, no untreated fellow eye (excluded from the OH incidence calculation) developed OH.

**Table 1:** Descriptive characteristics of children after cataract surgery and the incidence of ocular hypertension.

Characteristic	All included children		All included eyes	
	Total	Number of OH children (%)	Total	Number of OH eyes (%)
**Sex**				
Male	143	33 (23.1)	262	48 (18.3)
Female	63	13 (20.6)	117	18 (15.4)
**Cataract laterality**				
Bilateral	173	41 (23.7)	346	61 (17.6)
Unilateral	33	5 (15.2)	33	5 (15.2)
**Status after treatment**				
Aphakic eye	103	24 (23.3)	196	33 (16.8)
IOL eye	103	22 (21.4)	183	33 (18.0)
**Child’s age at surgery** *				
≤12 M	67	19 (28.4)	127	30 (23.6)
>12 M	139	27 (19.4)	252	36 (14.3)

* P<0.05;

OH = ocular hypertension; IOL = intraocular lens; M = months.

**Figure 2:**
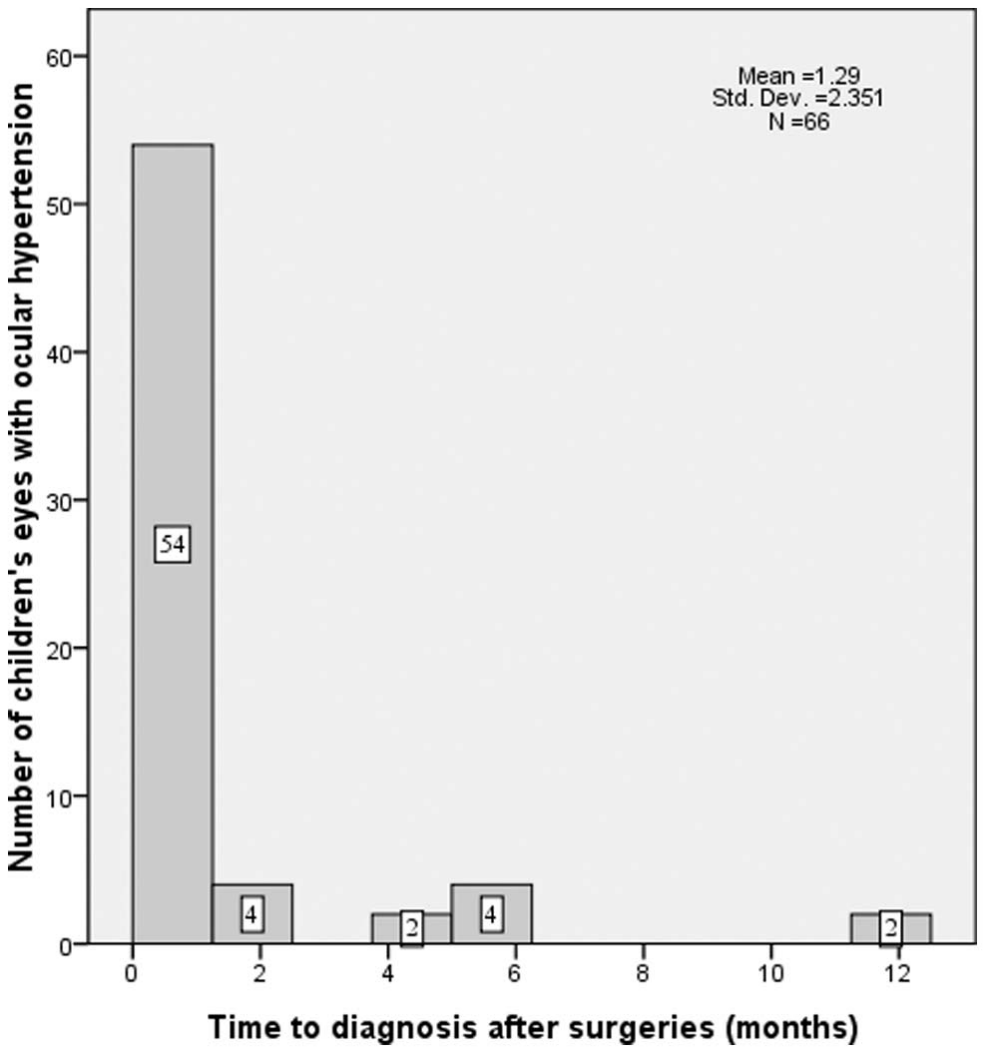
Postsurgery time to diagnosis of OH in children. OH onset (diagnosis) time after surgery was recorded as months. (OH = ocular hypertension).

The incidence of OH during the first-year follow-up period was 17.4% (66/379), which includes 33 of 196 (16.8%) aphakic eyes and 33 of 183 (18.0%) IOL eyes; there was no significant difference between the two groups (p = 0.53). The mean time between surgery and the final examination that included an IOP measurement was 14.0±3.2 months (median, 12 months; range, 10–16 months) for all of the analyzed patients’ eyes. OH was diagnosed at a mean of 1.3±2.4 months postsurgery (median, 0.25 months; range, 1 day-12.0 months; Figure 2) during the follow-up period. The peak onset times of OH were at the 1-week (34/66, 51.5%) and 1-month (14/66, 21.2%) follow-up appointments postsurgery. In all, 81.8% (54/66) of all OH patients were diagnosed within 1 month postsurgery.

**Figure 3:**
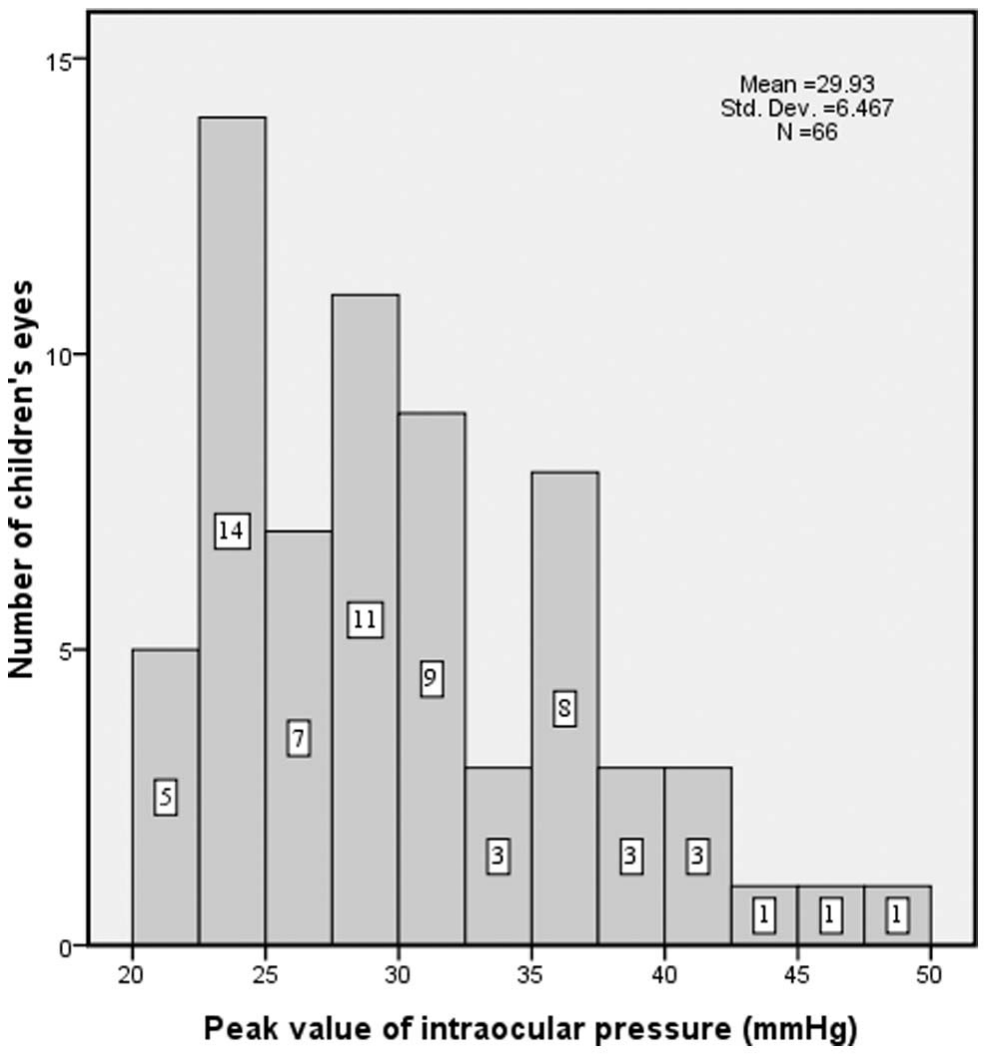
The peak IOP value at the time of OH diagnosis. The IOP value measured at the time of diagnosis was the highest IOP value in the OH-affected eyes during the study. (Notes: IOP = intraocular pressure; OH = ocular hypertension).

The mean IOP at the time of OH diagnosis was 29.9±7.5 mmHg (median, 29 mmHg; range, 21–48 mmHg). The IOP value measured at the time of diagnosis was the highest IOP value measured in the OH-affected eyes during the study. In other words, the average IOP value declined after the initial diagnosis. The IOP value in 41 of the 66 OH cases (62.1%) was ≤30 mmHg. The IOP value in 21 of the 66 OH cases (31.8%) was >30 and ≤40 mmHg. Only 4 OH cases (6.1%) had an IOP value >40 mmHg (Figure 3). The elevated IOP value of all OH eyes decreased to a normal value with our postoperative medicine administration regimen. None of the OH children, including the case that had an IOP of 48 mmHg, had symptoms in our study.

**Figure 4:**
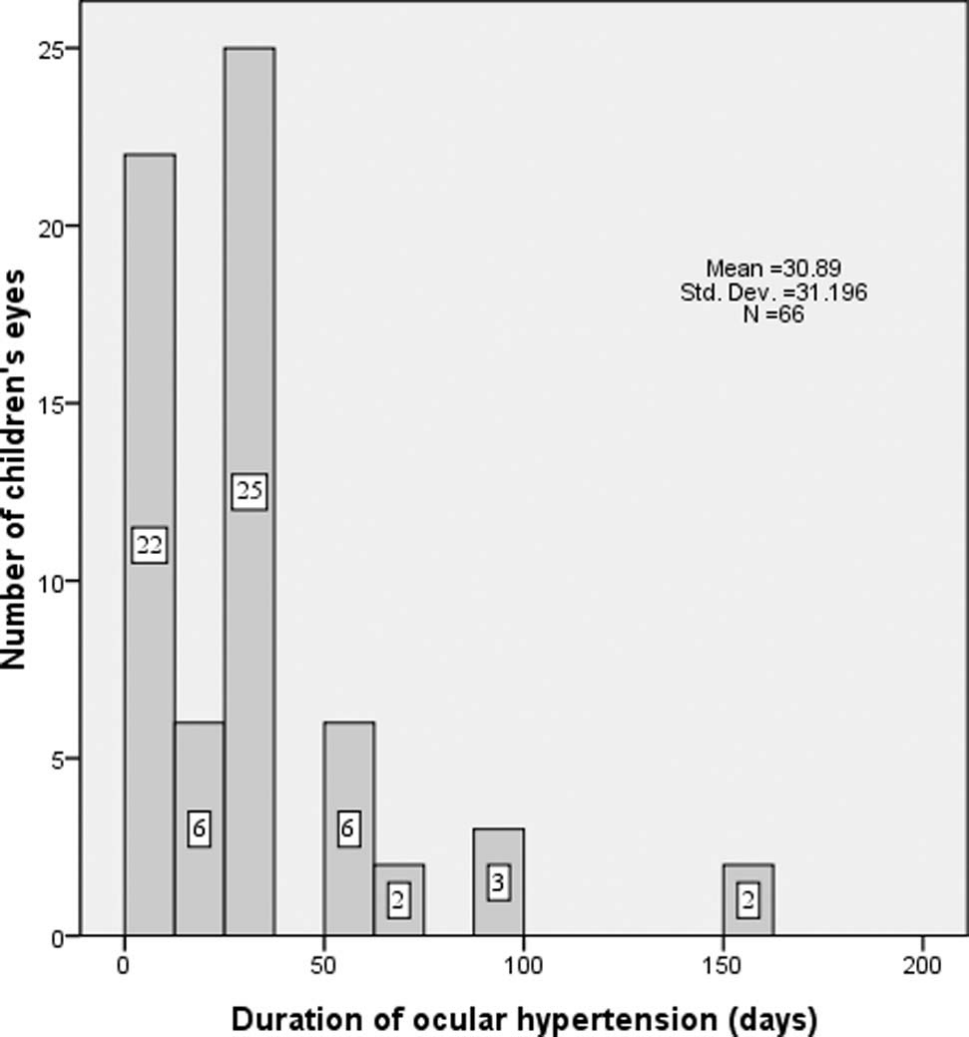
Duration between the appearance (diagnosis) and disappearance of ocular hypertension in children.

The duration of OH was 30.9±31.2 days (median, 30 days; range, 3–150 days). The elevated IOP persisted only 3 days in 13 OH eyes (13/66, 19.7%) after management. Fifty-one of the 66 OH cases (77.3%) had an OH duration ≤30 days. Only 2 cases with elevated IOP required 150 days (5 months) to recover under management (Figure 4). None developed a pupillary block with an iris bombé or uncontrollable OH after antiglaucoma medical management during the follow-up period.

After the OH was controlled for more than two weeks, the antiglaucoma medications were gradually discontinued. In the following 6 months, IOP fluctuation occurred when the antiglaucoma medications discontinued in 8 eyes (8/34, 23.5%) which were diagnosed as OH at the 1-week and 5 eyes (5/14, 35.7%) at the 1-month follow-up appointments postsurgery. In all, the 13 eyes with IOP fluctuation had a history of OH diagnosed within 1 month postsurgery (13/54, 24.1%). Before our data collection completed, 6 of these eyes with abnormal IOP fluctuation needed to use additional antiglaucoma medications for only 1 week. The other 7 eyes remained on antiglaucoma medications long term. However, no surgical management was needed for IOP control. No obvious change in optic nerve cupping or gonioscopy width was found. No abnormal elevated IOP was detected in the other 313 eyes (313/379, 82.6%).

## Discussion

Monitoring IOP to prevent secondary irreversible optic nerve damage is essential for pediatric cataract treatment, but the incidence and significance of the first-year elevated IOP to late-onset glaucoma is unclear [16]–[19]. Our ongoing prospective study seeks to determine the relationship of the incidence of OH in children during the first-year period and the risk of developing late-onset glaucoma. In this preliminary report, we define OH and present the first-year incidence as baseline characteristics of our ongoing long-term study.

In our study, the incidence of OH during the 12-month follow-up was 17.4% in 379 included eyes. These values are surprisingly low compared with the OH incidence that was reported in a study by Lam et al. [11], which is a comparable study of pediatric patients who were administered topical corticosteroids after strabismus surgery. The study by Lam et al demonstrated that 71.2% and 59.2% of children who were respectively administered topical 0.1% dexamethasone 4 times per day and 2 times per day after strabismus surgery developed IOP values greater than 21 mmHg at some stage of an 8-week follow-up period. The four-times-daily dosage group in the study by Lam et al had a similar postoperative corticosteroid regimen compared to that used in our study. However, there were several factors (e.g., the methods of IOP assessment, different surgical interventions and varying susceptibility for steroid use) that could cause differing IOP results between the two studies. In the Lam et al study, non-contact tonometry (XPERT NCT Plus, Leica, USA) was used for IOP assessment only in cooperative children, whose compliance with examination and unconscious blepharospasm would greatly affect the IOP and cause false positive results. In our study, all of the children, who were sedated or under general anesthesia, were assessed using a Tono-Pen tonometer (Reichert Inc., Seefeld, Germany), which is believed to be more accurate for IOP assessment. [16]–[18] In addition to the assessment methods, the differences in surgical intervention (i.e., intraocular vs. external eye surgery) may also be responsible for the discrepancy [19].

Varying susceptibility and medication adherence of topical steroids in different age groups could cause different IOP outcomes. [18], [20], [21] In our study, we determined that 12 months was the cut-off age (P<0.05) for the development of OH following surgery. 23.6% (30/127) of patients who were operated on at or before this age developed OH, whereas only 14.3% (36/252) of patients operated on after this age developed OH. This finding has been widely reported previously [1]–[3], [5], but in another five-year follow-up study in 2004, Peter found that 9 months may be the age threshold for the development of chronic glaucoma after pediatric cataract surgery. [22] The difference in the age threshold for developing acute OH or chronic glaucoma may be caused by different postoperative regimens and steroid-induced responses.

In our study, we determined that the peak OH onset times were at 1-week (34/66, 51.5%) and 1-month (14/66, 21.2%) follow-up appointments following surgery. Although most studies have reported that IOP increases 3 to 6 weeks after the introduction of topical steroid use, some elevation of pressure can be found in most patients as early as the first or second week of treatment. [24]–[28] In all, 81.8% (54/66) of patients with OH were diagnosed within 1 month postsurgery. However, 2 cases of OH were not diagnosed until 12 months postsurgery. It is suggested that there may be a process going on other than steroid-induced OH. Moreover, it is reported that the rate of progression from OH to aphakic glaucoma was 23% during a mean observation period of 7.2 years. [23] In our study, the mean time between surgery and the final examination that included an IOP measurement was 11±3.2 months (median, 12 months; range, 6–16 months) for all of the analyzed eyes, so we could only be able to determine this rate of progression in the OH-affected eyes of our prospective cohort study with longer follow-up period.

The clinical picture for infants with secondary glaucoma after cataract surgery resembles that of congenital glaucoma. [20] In our study, the mean IOP at the time of OH diagnosis was 29.9±7.5 mmHg (median, 29 mmHg; range, 21–48 mmHg). None of the OH children, including the case that had an IOP of 48 mmHg, had symptoms except OH in our study. These findings confirm the pediatric series reported by Yamashita et al., [29] in which none of the 5 OH patients had symptoms, including one who had an IOP of 47 mmHg. None of the patients developed a pupillary block with an iris bombé or uncontrollable OH after antiglaucoma medical management during the follow-up period. The duration of OH in our study was 30.9±31.2 days (median, 30 days; range, 3–150 days). Although in the following 6 months after the disappearance of the OH, IOP fluctuation occurred in 13 eyes with a history of OH diagnosed within 1 month postsurgery (13/54, 24.1%), no surgical management was needed for IOP control, and no obvious change in optic nerve cupping or gonioscopy width was found. Before our data collection completed, 6 of these eyes with abnormal IOP fluctuation only needed to use antiglaucoma medications temporarily for 1 week. The other 7 eyes remained on antiglaucoma medications long term. These clinical features demonstrate that the defining OH as a transient IOP rise post-operatively, which, whether related to surgical changes or steroid-induced outflow changes, resolves within a short time in most cases. However, the cause and significance of OH may be complex and multifactorial. Children who have suffered elevated IOP in the first-year period should be followed closely in our on-going prospective study to determine if there is an increased risk of developing late-onset glaucoma.

The results and interpretation of the present study must be understood within the context of its strengths and limitations. The study strengths include its large sample size, strict, regular follow-up protocol and consistent inclusion and exclusion criteria within the same research program (CCPMOH). Study weaknesses must also be acknowledged. Although this was a national program, all of the subjects underwent surgery and were followed at a single center without a randomized and controlled design for ethical reasons. Data regarding the morphologic classification of the cataracts were inconsistently available and excluded from this study. There were also variations in the surgical techniques. Optic nerve, retina thickness, gonioscopy, and visual field evaluations were inconsistently present in the records of these pediatric patients; consequently, we were unable to use these factors in the definition of glaucoma in this first-year study. However, further prospective observations of these subjects will allow us to include these missing data more consistently and determine whether the risk of developing glaucoma increases in subjects who have previously suffered acute and transitory OH during the first year postsurgery.

Despite these limitations, this study remains one of the first ongoing prospective studies with large sample size and long-term follow-up to determine the incidence of first-year OH in children after cataract surgery. Our first-year report also provides a cohort of specific children with defined baseline characteristics for our ongoing prospective cohort study to ultimately address the possible relationship between the first-year elevation in IOP and late-onset glaucoma.
